# Deposition Offset of Printed Foam Strands in Direct Bubble Writing

**DOI:** 10.3390/polym14142895

**Published:** 2022-07-16

**Authors:** Prasansha Rastogi, Cornelis H. Venner, Claas Willem Visser

**Affiliations:** Engineering Fluid Dynamics Group, Department of Thermal and Fluid Engineering, Faculty of Engineering Technology, University of Twente, P.O. Box 217, 7500 AE Enschede, The Netherlands; p.rastogi@utwente.nl (P.R.); c.h.venner@utwente.nl (C.H.V.)

**Keywords:** Direct Bubble Writing, foams, deposition offset, 3D printing, photopolymerization

## Abstract

Direct Bubble Writing is a recent technique to print shape-stable 3-dimensional foams from streams of liquid bubbles. These bubbles are ejected from a core-shell nozzle, deposited on the build platform placed at a distance of approximately 10 cm below the nozzle, and photo-polymerized in situ. The bubbles are ejected diagonally, with a vertical velocity component equal to the ejection velocity and a horizontal velocity component equal to the motion of the printhead. Owing to the horizontal velocity component, a discrepancy exists between the nozzle trajectory and the location of the printed strand. This discrepancy can be substantial, as for high printhead velocities (500 mm/s) an offset of 8 mm (in radius) was measured. Here, we model and measure the deviation in bubble deposition location as a function of printhead velocity. The model is experimentally validated by the printing of foam patterns including a straight line, a circle, and sharp corners. The deposition offset is compensated by tuning the print path, enabling the printing of a circular path to the design specifications and printing of sharp corners with improved accuracy. These results are an essential step towards the Direct Bubble Writing of 3-dimensional polymer foam parts with high dimensional accuracy.

## 1. Introduction

**Figure 1:**
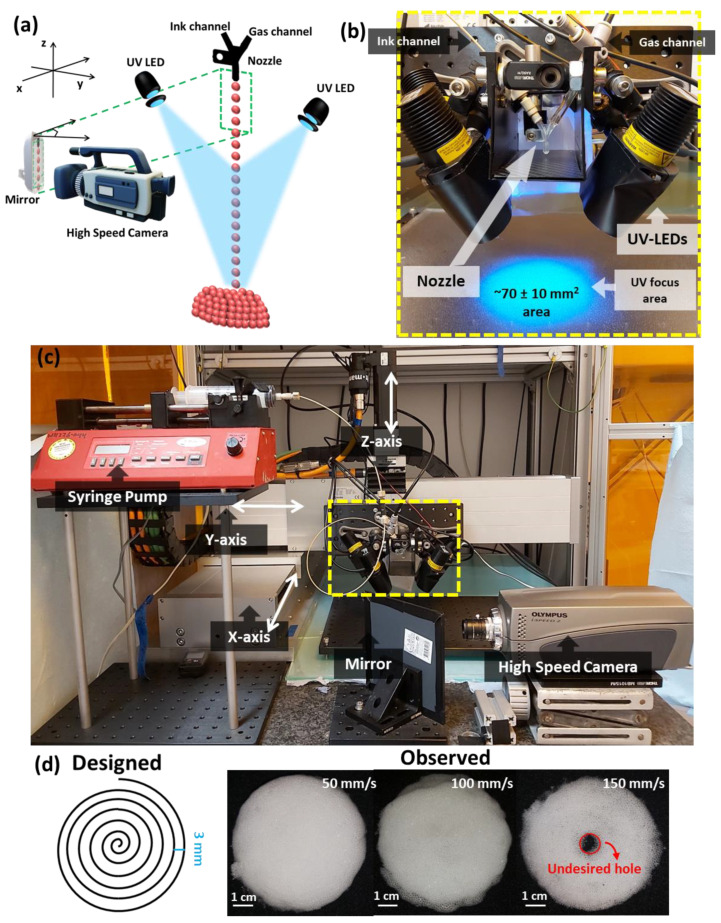
Overview of Direct Bubble Writing (DBW). (**a**) Schematic representation of the DBW setup. Bubbles flow from the nozzle and are captured by a high-speed camera. Down-stream, these bubbles are photopolymerized by 4- UV-LEDs, yielding a foam construct shown in red. (**b**) Photograph of the printhead, with key elements as indicated. (**c**) Overview of the printer. The dashed area is magnified in (**b**). (**d**) The designed pattern (left) was printed at three different velocities of the printhead. For high velocity (150 mm/s), the print showed an unfilled region at the center of the disk. Reducing the printhead velocity mitigated this issue. Samples were printed with a printhead acceleration a = 1 m/s^2^, a gas pressure P = 7 kPa, and a flow rate of the resin Q = 15 mL/min.

Direct Bubble Writing (DBW) is a recent technique for printing of 3-dimensional (3D) foam architectures with control over the local density and architecture of the foam structure [1]. The distinctiveness of DBW lies in its throughput (10 g/min), a wide range of printing speeds (up to 500 mm/s), tunability of the local physical and morphological properties of the foam (porosity, polydispersity, pore size, and density), design freedom, and single-step processing of the printed parts [1]. DBW relies on making bubbles that consist of photocurable resin one-by-one. The bubbles are formed by flowing the resin through the shell of a core-shell nozzle and a gas through the core. The bubbles are exposed to UV light during their impact, resulting in the partial solidification in the air by photopolymerization and complete curing within 0.2 s after impact onto the build platform. As a result, this technique enables the printing of shape-stable pillars and out-of-plane foam printing designs, including angular pillars, horizontal overhangs, and inverted ‘V’ shapes, all without any external supports. The local density of the foam properties can be modulated by the input pressure of the gas onto the core-shell nozzle [1,2,3]. This ability to *locally* control the properties of cells in the foam extends extrusion-based methods for the additive manufacturing of foams [4]. DBW classifies as a material jetting technique, where a microfluidic core-shell nozzle is used to generate air-filled droplets. A scheme of DBW is shown in Figure 1a.

In DBW, the ejected bubbles fly from the nozzle to the build platform in an approximately diagonal trajectory. Their initial vertical velocity component is determined by the ejection velocity, $v_{j,0}$, and the initial horizontal velocity component equals the velocity of the printhead, $v_{p,0}$. As a consequence of this horizontal velocity component, the impact location of a bubble differs from its ejection location. This generates dimensional discrepancies between the designed and the final printed part. Up to now, the printing speed was restricted to 100 mm/s to limit these dimensional discrepancies [1,2].

As known from previous work on inkjet and binder jetting [5,6,7,8], this offset between the expected and realized droplet position is a function of the printhead velocity [9,10,11]. Especially fine details including patterns, slots, or holes are compromised by this offset [12,13]. To improve the predictability of the droplet impact position in drop-on-demand printing, the moment of droplet generation was shifted in time [10], and applied to improve the printing of straight lines, corners, and curves [10,14]. Additional constraints were required to limit the aerodynamic effects, such as limiting the nozzle-substrate distance to around 2 mm for small (50 μm diameter) droplets [6,15,16,17]. 

However, modulating the moment of ejection is not possible for DBW since bubbles are created as a continuous stream. Therefore, here we analyze the print path and propose modifications to obtain improved dimensional accuracy. The materials and methods are described in Section 2, followed by calculations and measurements of the offset of the print path in Section 3 and the conclusion in Section 4.

## 2. Materials and Methods

### 2.1. Materials

Poly(ethylene glycol) diacrylate (PEGDA, number-averaged molecular weight of 700 g/mol and density 1120 kg/m^3^ at 25 °C) and Diphenyl(2,4,6-trimethylbenzoyl)phosphine oxide (TPO) were purchased from Sigma-Aldrich, Schnelldorf, Germany. Furthermore, a viscous non-ionic surfactant (Tween20, with a critical Micelle Concentration of 0.06 mM at 20 °C) and demineralized (DM) water were used.

### 2.2. Preparation of the Ink

All preparation of the ink for the DBW of foam was carried out in a dedicated lab with yellow lighting, preventing any light-induced initiation of the reaction. To prepare the ink, PEGDA and DM water were mixed in a 1:1 ratio (50 g of each). Then, 2 wt% Tween20 was added to the PEGDA:water mixture (2 g) and this mixture was gently stirred to homogenize the resulting solution. Later, 1 wt% TPO photo-initiator (with respect to the PEGDA amount) (0.5 g) was added to the ink. The ink was magnetically stirred overnight in a closed, light-shielded vessel to dissolve the TPO, resulting in a clear ink with a high polymerization reactivity when exposed to UV light. This ink was purged with nitrogen just before use, to remove any oxygen that could inhibit the photopolymerization reaction. During printing, the ink bubbles were exposed to UV light. For experiments that did not require curing, water + Tween20 (2 wt%) mixtures were used as a model system.

### 2.3. Printer Setup

A scheme of the DBW is shown in Figure 1a. The printer setup consists of a 3-dimensional automated stage, on which a printhead is mounted (Figure 1b; an overview of the entire printer is shown in Figure 1c). The printhead carries a nozzle for producing bubbles, a low speed camera to observe the bubble formation, and UV LEDs for polymerization. Below the printhead, a flat build platform is located.

A core-shell nozzle was 3D-printed on a FORM3 printer (FORMLABS), using Clear resin V4 and a resolution setting of 25 μm. The core nozzle was supplied with gas (compressed air or nitrogen) using a calibrated pressure controller (EL-Press P-602CV (p2-Control), Bronkhorst). The shell of the nozzle was provided with the ink via a computer-controlled syringe pump (NE-8000) driving a 60 mL plastic syringe filled with ink. A Luer lock and hollow PEEK tubing (1 mm inner diameter) (IDEX Health and Science) were used to connect the syringe to the nozzle. 

The ejected stream of monodisperse bubbles was illuminated from an intensity-tunable green light (SP-02-L17 star LED, Luxeonstar) powered by a 32 V power supply (Figure S1a). Electronics for LEDs were custom made. The bubble stream was visualized using a low-speed IDS camera (UI-3240LE-M-GL (AB00426)) fixed on the printhead (Figure S1b). Alternatively, the translation of each bubble and corresponding horizontal motion was tracked using a high speed camera (Olympus i-speed 2 CDU). A frame rate of 2000 fps with a shutter duration of 50 μs were selected. Determination of the bubble trajectory was based on 1300 frames. A bright light source (LAvision) was used for brightfield illumination (Figure S1c). To fit the high-speed imaging setup within the closed printer cabinet, a mirror was placed to minimize the footprint (Figure 1a,c).

The bubble stream was polymerized upon impact using 4 monochromatic UV LEDs (2-M365L2 and 2-M375L4, ThorLabs) that were controlled by a 24 V power supply, enabling the rapid photopolymerization of inks containing either Li-TPO (with a 365 nm absorption peak) or TPO (with a 375 nm absorption peak) as a photo-initiator. The UV light was focused onto the build surface (~70 ± 10 mm^2^ area) and centered on the bubble stream using a convex lens for each UV LED (ACL25416U-B, Thorlabs).

The motion of the nozzle in x−y−z space, the input gas pressure, and the ink flow rate were controlled using Motion Perfect software. Upon starting the printer motion, the UV LEDs were manually switched on for each experiment. The resulting foam samples were removed from the build platform by mild scraping and collected for further optical characterization. Due to mechanical constraints, the printing of corners sometimes led to a printer crash. The printer working range was measured as a function of the velocity and the corner angle, as shown in Figure S2. Based on this result, the printing of 90° corners was limited to printhead velocities below 200 mm/s.

The composition of the ink, the ink flow rate Q, the gas encapsulated in core, the gas pressure P, the printhead velocity $v_{p}$, the acceleration a, the distance between the nozzle tip and build platform h, and the printhead trajectory dimensions are shown in Table 1.

**Table 1:** Summary of the control parameter settings.

Control Parameter	Figure 2 (Trajectory)	Figure 3 (Circle Pattern)	Figure 3 (Square Pattern)
**Ink composition**	Water + Tween20 (2%)	PEGDA700 + water (1:1) + Tween20 (2%) + TPO (1%) (w.r.t PEGDA)	PEGDA700 + water (1:1) + Tween20 (2%) + TPO (1%) (w.r.t PEGDA)
**Ink Flow Rate (mL/min)**	12	15	15
**Gas in core**	Compressed Air	Nitrogen	Nitrogen
**Gas Pressure (kPa)**	3.5	7	7
**Printhead Velocity (m/s)**	0.1	0.05, 0.1, 0.2, 0.3, 0.4, 0.5	0.05, 0.1, 0.15, 0.2
**Printhead Acceleration (m/s^2^**)	1	1	1
**Nozzle-to-build** **platform distance (m)**	0.1	0.1	0.1
**Printhead trajectory** **dimensions (m)**	0.1 m	Diameter = 0.06 m	Side length = 0.06 m

**Figure 2:**
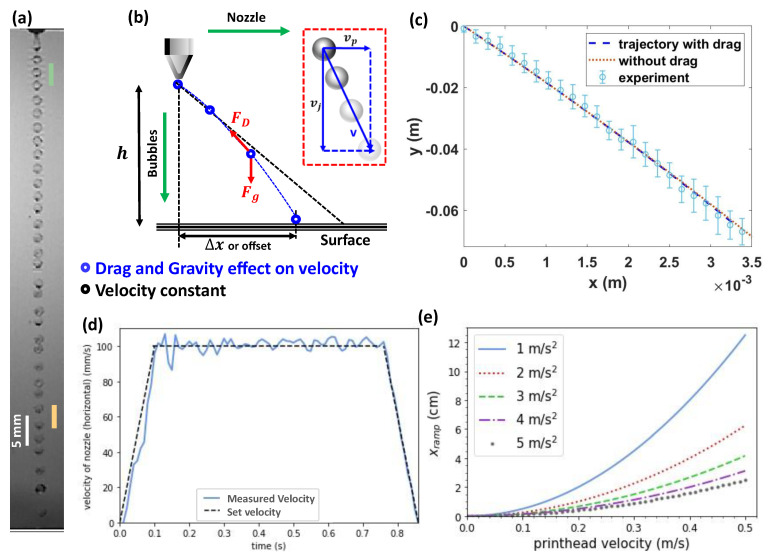
Characterization of the DBW process. (**a**) High speed video frame displaying stable ejection of a monodisperse bubble stream from the nozzle. The bubbles are ejected downwards with a velocity of 1.79 ± 0.11 m/s. (**b**) Example path of the bubble stream. Bubble deposition occurs at a distance Δx away from the ejection location. The red arrows represent the drag force and the gravity force acting on the bubbles in–flight. The black dotted line shows the trajectory of the bubbles without the influence of drag or gravity. Inset: Definition of the velocity components. (**c**) Measured trajectory of the bubbles after ejection from the nozzles. The error bars show the standard deviation of at least 16 tracked bubbles. The blue dashed and orange dotted lines indicate the calculated trajectory taking into account both drag and gravity and only gravity, respectively. The measurements are taken for *Q* = 12 mL/min, *P* = 3.5 kPa, and $v_{p}=$ 100 mm/s. (**d**) The printhead velocity as a function of time. The black dotted line shows the set velocity for a= 1 m/s^2^. The blue line shows the measured printhead velocity. (**e**) The calculated ramp distance traveled before attaining a constant velocity of the printhead, as a function of the velocity and acceleration.

**Figure 3:**
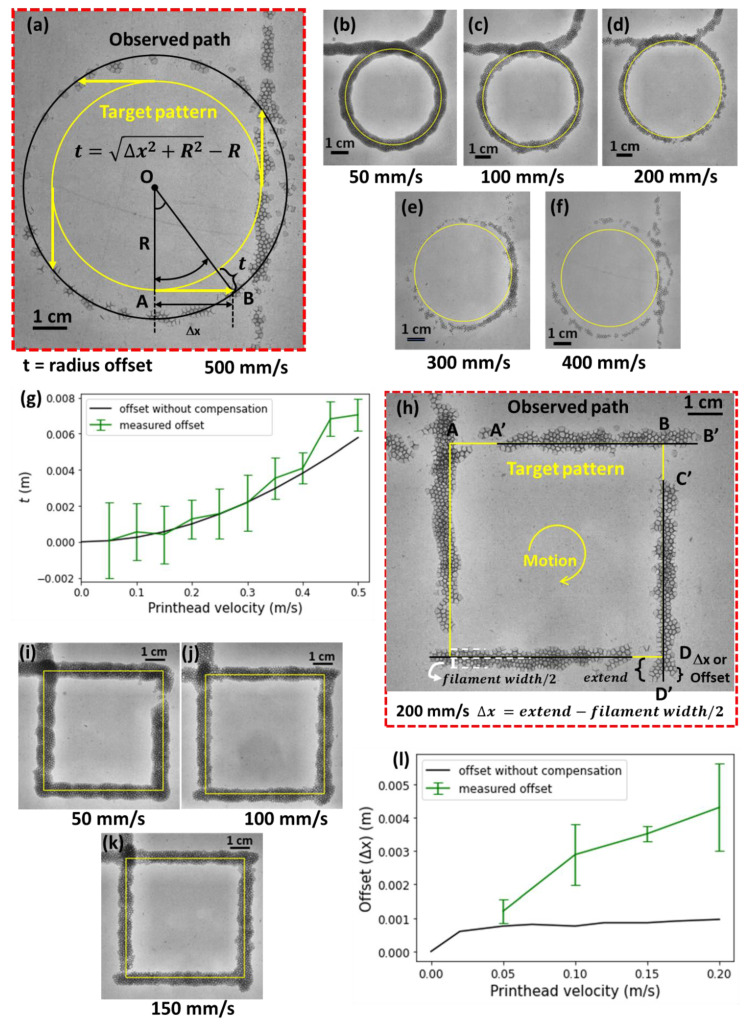
Observations and model results of the printed foam strands. (**a**) The observed printed bubbles stream (photo) for a printhead velocity of 500 mm/s. The yellow circle shows the preset circular nozzle path of 60 mm diameter while the black line shows the expected location of the printed foam strands. (**b**–**f**) Printed circles for increasing printhead velocities. (**g**) The radial offset, t as a function of the printhead velocity. The green line shows measured mean values and the black line represents Equation (8). (**h**) The deposited bubble for a square geometry printed with a nozzle head velocity of 200 mm/s. The offset is shown as Δx, which was determined by extension beyond the yellow line (modeled path) after compensating the print thickness in the direction of motion of the printer. (**i**–**k**) Printed square patterns for increasing printhead velocities. (**l**) The measured and modeled offset Δx as a function of the printhead velocity for sharp corners. The error bars in (**g**,**l**) indicate the standard deviation of the offset, based on thickness measurements.

### 2.4. Image Processing

Videos from the high speed camera were post-processed using a script built in Python. From two consecutive frames, the translation of the bubbles was determined using a home-made cross-correlation tool in x and y directions. Further details on the image processing methods are provided in Supplementary Information (text, Figures S3 and S4). 

To verify both the horizontal velocity of the printhead and the calibration of our Matlab script for image processing, the preset nozzle velocity is compared to the measured value. For the observed linear trajectory, the nozzle head exhibited acceleration followed by a constant velocity, and deceleration until the printer stopped. For a set velocity of 100 mm/s, the printhead movement registered an average velocity of 100.7 ± 3.4 mm/s, as shown in Figure 2d. The set acceleration and deceleration values of 1 m/s^2^ are reasonably well-recovered. The ramp time (during which the nozzle accelerates or decelerates) is provided by:

(1) $$t_{ramp}=\frac{v_{p,end}-v_{p,0}}{a}$$

with $v_{p,end}$ and $v_{p,0}$, the final and initial nozzle head speed, respectively, and a the acceleration. The associated ramp distance traveled by the printhead before attaining a constant velocity is:

(2) $$x_{ramp}=\frac{v_{p,end}^{2}-v_{p,0}^{2}}{2a},$$

as shown in Figure 2e.

Measuring the bubble motion at a constant velocity of the printhead was ensured by adding these ramp distances to the print path (extra path of 2 × $x_{ramp}$) and measuring at the constant velocity region.

### 2.5. Cyclic Compression Testing

Three cylindrical 3D foam samples (60 mm diameter, 15 mm height) were printed according to the specifications described in the norm EN ISO-3386-1 for compressive testing [18,19]. These samples were deformed from 0% to 80% (in steps of 10%) of their initial height in a compression testing machine (Zwick/Roell Z5.0) at a strain rate of 50 mm/min using a load of 2.5 kN. Stress–strain diagrams were obtained at each deformation. In the initial cycle, the sample was compressed to a 10% strain and unloaded. After 10 cycles, the strain was set to 20%. This procedure was repeated up to a strain of 80%.

## 3. Results

Bubbles were ejected from the printhead following a spiral print path with a maximum diameter of 60 mm, as shown in Figure 1d. However, as shown in the observed results (Figure 1d), the deposited bubble stream did not land in this programmed spiral pattern. We hypothesize that the difference between the ejection location and the deposition location is caused by the horizontal component of the bubble’s velocity, resulting in an offset Δx [10]. 

Therefore, we first assess the bubble trajectory. Figure 2a depicts the stream of monodisperse bubbles produced from the water + tween mixture as captured by the high-speed camera. During flight, gravity and drag act on the bubbles. Here, the gravitational force is $F_{g}=mg$, with m the mass of the bubble and g=−9.81 m/s^2^ the gravitational acceleration. The drag force is provided as $F_{D}=\frac{1}{2}\rho_{air}C_{D}Av^{2}$, with $C_{D}=\frac{24}{Re}\left(1+1.15Re^{0.687}\right)$ the drag coefficient, $Re=\frac{\rho_{air}D\left|v\right|}{\mu_{air}}$ the Reynolds number, $\mu_{air}=$ 1.825*10^−5^ kg/m.s the dynamic viscosity of the air, $\rho_{air}=1.2$ kg/m^3^ the density of the air, $A=\pi {\left(\frac{D}{2}\right)}^{2}$ the frontal area of the bubble, D the outer diameter of the bubble, and v $=\left(v_{p},v_{j}\right)$ the velocity vector. Now, using ΣF=ma, with a the acceleration of the bubble, we obtain $ma_{x}=F_{D,x}$ and $ma_{y}=F_{g}+F_{D,y}$ for the forces in x-directions and y-directions, respectively. The solution of these equations is implicit as the velocity is required to determine $F_{D}$. Therefore, a solver was programmed in Matlab to obtain bubble trajectories for times 0<t<0.5 s. The resulting calculated bubble trajectories are essentially equal with and without drag for bubbles with a diameter of 1.6 mm and liquid fraction of 0.2, as shown in Figure 2c (the corresponding forces, velocities, and Reynolds numbers are shown in Figure S5). Therefore, for the limited range of the bubble trajectory in DBW, we ignore the drag force. 

Figure 2b shows the offset Δx between the ejection location and impact location. Assuming a separation distance h between the nozzle and the surface we obtain:

(3) $$v_{j,f}=\sqrt{v_{j,0}^{2}+2gh}$$

$v_{j,f}$ is the velocity of bubble at distance h from nozzle. The time, $t_{im}$, between ejection and impact on surface is then provided by:

(4) $$t_{im}=\frac{-v_{j,0}+v_{j,f}}{g}$$

The displacement of the droplet from the ejection location to the deposition location Δx is then provided by:

(5) $$\Delta x=t_{im}\times v_{p}$$

where we assume constant $v_{p}=v_{p,0}$. This offset Δx will be inherited in the design for the corresponding value of $v_{p,0},\;v_{j,0},$ and h, by integrating Equations (3)–(5) into Equation (6).

(6) $$\Delta x=v_{p,0}*\left(\;\frac{-v_{j,0}+\sqrt{v_{j,0}{}^{2}+2hg}}{g}\right)$$

Figure 3a shows how this offset Δx results in a major difference between the printhead trajectory (in yellow) and the printed path (in black). After transiting one revolution along the print path, the end points of all tangential offsets (4 yellow arrows shown), therefore, result in a circle with a larger diameter as compared to the printhead trajectory. The radius, OB, of this circle is provided by Equation (7), resulting in a radius offset t as given in Equation (8).

(7) $$OB=\sqrt{AB^{2}+OA^{2}}$$

t=OB−OA,

thus,

(8) $$t=\sqrt{\Delta x^{2}+R^{2}}-R$$

where OA=R (radius of the circle), AB= tangential offset Δx, and OB=R+t.

The bubble deposition location was assessed as a function of the printhead velocity for 50 mm/s < $v_{p,0}$ < 500 mm/s in Figure 3a–f. The printhead followed a circular path with a diameter of 60 mm, as indicated by the yellow circles. The modeled (black) and the experimental (in green) radius offset were in reasonable agreement, as shown in Figure 3g. 

Another essential design element when prototyping new parts is sharp corners [14,20]. Therefore, a square pattern with 90° angles was printed, as shown in Figure 3h. Here, the nozzle follows trajectory AB, whereas deposition appears at a shifted position, A’B’. Photographs of squares printed at velocities from 50 mm/s to 200 mm/s are depicted in Figure 3h–k. For a printhead speed of <100 mm/s, the offset was smaller than the printed foam strand width itself. With progression in velocity until 200 mm/s, the offset became noticeable and it also led to the discontinuous writing of bubbles near corners. The measured offset is shown as a function of the printhead velocity in Figure 3l.

The shifted deposition location was modeled in MATLAB by calculating the expected landing position of bubbles ejected at different instants, for the decelerating (and accelerating) printhead. The results of this model are shown in Figure S7. Figure S7a shows the printhead location for times separated by a time step Δt= 1 ms; the corresponding deposition location of the printed foam strands is shown in Figure S7b. The observed print patters is shown in Figure S7c,d, revealing an offset as well as a gap between the printed line segments. This large offset and gap was not predicted by our model, which assumes that the bubbles are launched from a perfectly decelerating and accelerating printhead. Strong vibrations of the printhead when plotting sharp corners were observed in high-speed videos; such gaps have also been known to occur in inkjet printing [14]. Additionally, these vibrations intensified with increasing printhead velocity, which forced the bubbles to fly farther away before deposition and, therefore, this offset mismatch was observed. Modeling these vibrations extend the scope of this article, but clearly a solution must be found if structures with thin walls and sharp corners are required.

**Figure 4:**
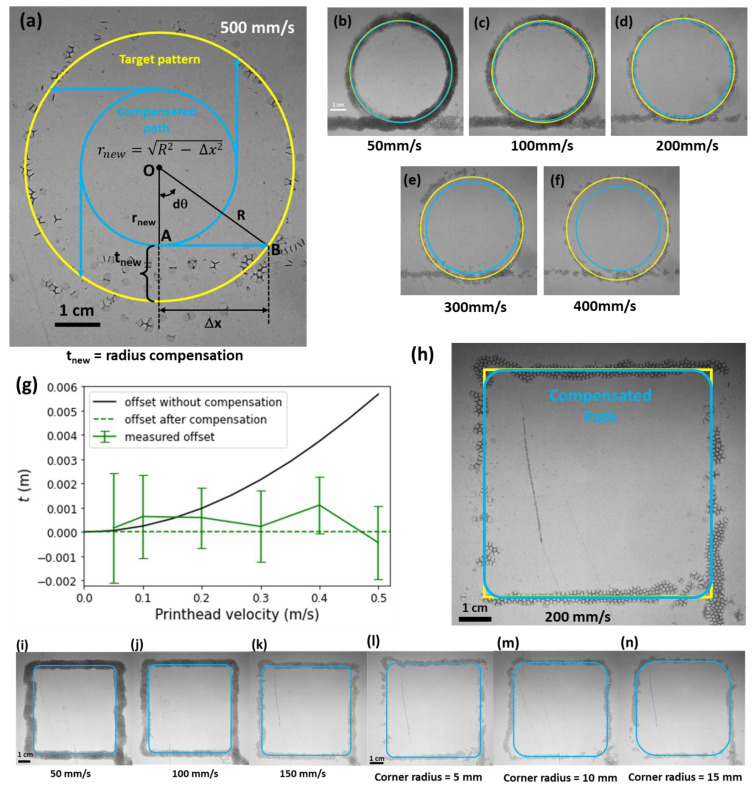
Print path compensation scheme to alleviate the generated offset at different printer velocities. (**a**) Photo of the target circular pattern with a radius R = 30 mm (in yellow), the observed printed foam strands (photo), and the compensated path of the printhead to obtain this target. The printhead velocity was set to $v_{p}$ = 500 mm/s. (**b**–**f**) Printed patterns (photos) match the target diameter (yellow line) by adjusting the printhead trajectory to a calculated path (blue line) for velocities as indicated. (**g**) Measured offset (markers) compared to the target (red dashed line). The black line shows the modeled offset without compensation. (**h**) Photo of a foam strand in a compensated pattern with corner radius of 5 mm, for a printhead velocity, $v_{p}=$ 200 mm/s. (**i**–**k**) Printed rounded squares for velocities from 50 mm/s to 200 mm/s (in (**h**)). (**l**–**n**) Printed patterns for increasing corner radii. The control parameters of these prints were Q = 15 mL/min, P = 7 kPa, and a = 1 m/s^2^.

To improve the geometrical and dimensional integrity for an increased range of printhead velocities, we propose a compensation method that exploits the above-described offset model. We define a target radius for the printed circular pattern (the yellow line) in Figure 4a and adjust the path of the printhead to obtain this pattern (the compensated path in Figure 4a, shown in blue). The compensated path was derived (blue circle) following Equation (8).

(9) $$OB^{2}=OA^{2}+AB^{2}$$

Contrarily, OA in Equation (9) became the new radius (after compensation), $r_{new}$, AB is the offset, Δx, which was determined from Equation (5) for a specific $v_{p,0}$ and OB became the radius, R, that was to be printed (e.g., 30 mm). Rearranging and substituting the respective values in Equation (9) would render the radius of the compensated circle in Equation (10).

(10) $$r_{new}=\sqrt{R^{2}-\;x^{2}}$$

Equation (10) was implemented in the print path to determine the diameter of the compensated circle $2\times r_{new}$ at printhead velocities from 50 mm/s to 500 mm/s, as depicted in Figure 4b–f. Figure 4g shows the measured circle radius (green markers) against the desired circle (red dotted line), showing that the error is diminished as compared to using the printhead path without the compensation black line, as discussed above). 

For square corner designs, the print path was compensated by rounding the corner as shown for a printhead velocity of 200 mm/s in Figure 4h. Similar results for the velocities 50 mm/s to 150 mm/s are shown in Figure 4i–k. As compared to Figure 3h–k, the deviation and continuity of the print at similar velocities were improved. A multi-layered structure with rounded corners is shown in Figure S8c,d. In comparison to the same construct with sharp corners (Figure S7c,d), the gaps between the walls are prevented. However, a remaining deviation at the corner was inherent due to the radial offset (analogous with the circular designs). This deviation was further reduced by incrementally adjusting the radius at corners as shown in Figure 4l–n. These rounded designs were printed at a 200 mm/s printed speed with 5 mm, 10 mm, and 15 mm corner radii, respectively.

**Figure 5:**
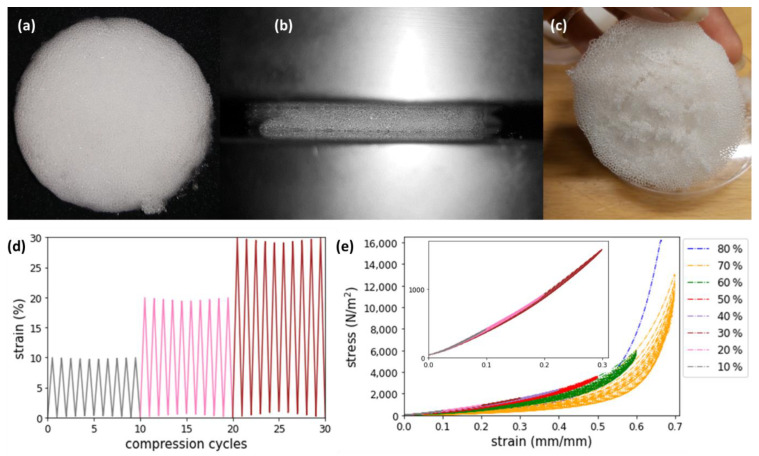
Compression behavior of printed foams samples. (**a**) Cylindrical samples with a diameter of 60 ± 1 mm and thickness of 14 ± 1 mm are used for compression testing. (**b**) Sample under 70% compression in the testing machine. (**c**) Sample after compressing to 80%, showing loss of integrity. (**d**) Loading as measured during cyclic mechanical testing. Compression cycles are shown for the strain values of 10%, 20%, 30%. The colors are indicated in the legend of (**e**). (**e**) Stress–strain curves for the compressive loading up to 80% strain. The average measurement of 3 samples is shown. Hysteresis is almost negligible up to 40% strain (the inset details the strain curves between 10% and 30%).

Finally, 3-dimensional foam samples were printed and tested for cyclic compressive loading from 0–80% strain as shown in Figure 5. The cylindrical sample printed in the form of a cylinder (Figure 5a) was compressed to 80% strain between the plates of a compression testing machine (Figure 5b). At 80% compression, all the foam cells were fractured, and the sample was completely destructed, as shown in Figure 5c. The compression testing was performed for 10 cycles at a single strain after which the strain was incremented in steps of 10%, as shown in Figure 5d, for an initial 30 cycles. Negligible hysteresis was observed until 40% strain (Figure 5e-inset), reflecting the elastic deformation of the foam cell walls by bending, buckling, or stretching. Beyond 40% strain, significant hysteresis was observed (Figure 5e). A sharp increase in stress and widening of the hysteresis curve was visible for 70% strain, reflecting densification and plastic deformation of the cell walls [21,22,23,24,25,26]. Most significantly, our observations show that a closed-cell foam of a brittle material (for example, PEGDA disks shatter when dropped on the ground) absorbed energy efficiently and reversibly up to 40% compression. 

## 4. Conclusions

Foam strands were printed to quantitatively assess the effect of the printhead velocity on the designed dimensions for the DBW of photosensitive diacrylate resins. The printing offset was assessed as a function of the printhead velocity for circular and square patterns. Pronounced discrepancies between the trajectory of the nozzle and the printed bubble stream were observed, especially for printhead velocities >100 mm/s. The mismatch was modeled by describing the motion of the ejected bubbles in time and space and measured by analyzing printed foam patterns. Subsequently, a compensation scheme consisting of adjustments in the dimensions of the to-be-printed architectures was applied, resulting in patterns matching the intended dimensions with an error smaller than the foam strand width. This compensation scheme is an essential step towards dimensionally accurate 3D printing of foam parts with DBW. 

## Data Availability

Data is contained within the article or supplementary Material.

## Supplementary Materials

The following supporting information can be downloaded at: https://www.mdpi.com/article/10.3390/polym14142895/s1, Figure S1: LED sources used for imaging with low-speed camera and high-speed camera in direct bubble writing set up.; Figure S2: Operating window of the printer as a function of the corner angle and printhead velocity.; Figure S3: Cross-correlation tool for image processing to calculate bubble velocity.; Figure S4: Image processing and circle fit on the printed foam circular samples.; Figure S5: Theoretical analysis of the drag and gravity forces and their influence on the flight of a single bubble.; Figure S6: Velocity profile of bubbles in motion as a function of the distance travelled from the nozzle.; Figure S7: Model and measurements of the printed bubble stream for sharp corners.; Figure S8: Model and measurements of the printed bubble stream for round corners.
