# Supplementary material for: Deposition Offset of Printed Foam Strands in Direct Bubble Writing
Source: Polymers (Basel). 2022 Jul 16;14(14):2895. doi: 10.3390/polym14142895 (PMC9321078; doi:10.3390/polym14142895)
Supplement: Supplementary file 1 [file polymers-14-02895-s001.zip › polymers-1774944-supplementary.pdf]

Supplementary Information

Deposition Offset of Printed Foam Strands in Direct Bubble Writing

Prasansha Rastogi, Cornelis H. Venner and Claas Willem Visser *

Engineering Fluid Dynamics Group, Department of Thermal and Fluid Engineering, Faculty of Engineering Technology, University of Twente, P.O. Box 217, 7500 AE Enschede, The Netherlands;
p.rastogi@utwente.nl (P.R.); c.h.venner@utwente.nl (C.H.V.)
* Correspondence: c.visser@utwente.nl

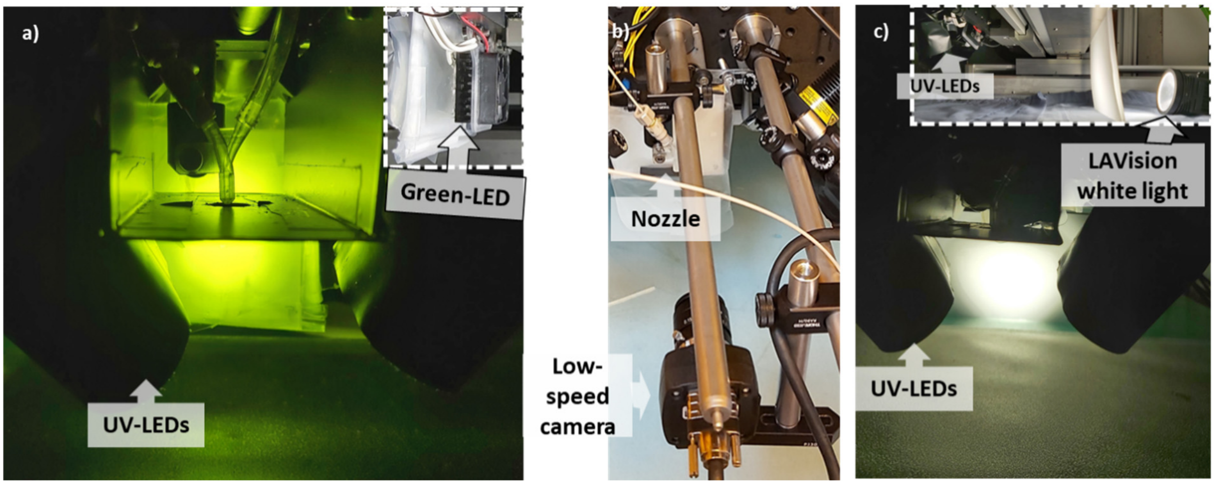

Figure S1: LED sources used for imaging with low-speed camera and high-speed camera in direct bubble writing set up. a) Green light sources (tunable intensity) mounted behind the nozzle (inset) to visualize bubble formation in the camera from the front. b) Showing the low-speed camera mounted on printhead to visualize bubble formation during printing (used with green light in a)). c) Brightfield LAVision light illuminated from behind the nozzle. This light is kept at a distance to prevent over-illumination of region of interest due to fixed light intensity (inset).

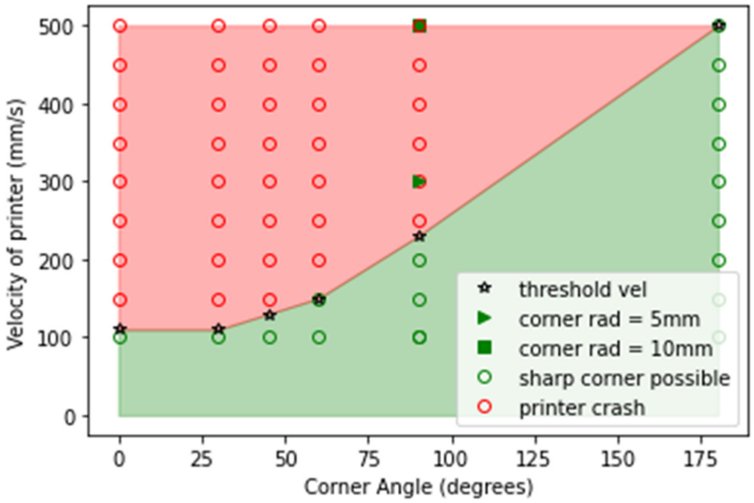

Figure S2: Measure operating window of the printer as a function of the corner angle and printhead velocity, for an acceleration of 1m/s^2. Green circles represent successful tuning of the printhead; red circles indicate a printer crash. Black stars represent velocity that could be printed. The green area serves as a guide to the eye for the operating window. Solid triangle and square points represented that changing sharp corner to round corner (of radius 5mm and 10 mm), printability of 90° angle could be extended to 300mm/s and 500mm/s (maximum printer speed) respectively.

Image Processing

To execute the analysis for determining bubble velocity in python, different libraries e.g. opencv (cv2), numpy, pandas, and matplotlib.pyplot (plt) were utilized. To execute cross-correlation tool, a window of 50*50 pixels was retrieved at breakup point of bubble (where it completely detached from nozzle) (5 mm below nozzle head) (Figure SI3(a)) and near end of frame at bottom (~65 mm down from nozzle head) in both the adjacent frames (intensity matrix). These images, after some pre-processing (Figure SI3(b)), were then reformed into binary images using Otsu thresholding (Figure SI3(c)) which aided in later cross-correlation calculations between 2 frames. The first binary image in the pair of images was scanned pixel by pixel in row and column over itself (case 1) and over the other image (case 2) to detect equivalence between 2 frames. The maxima were established when, for either case, an image overlapped with its pairing/counter image or maximum matching between images were achieved. The coordinate for this maximum correlation was realized at center for case 1 while it was repositioned for case 2. This shift in the peak position deduced the translation in bubbles between the two frames in row and in column (Figure SI3(d)). However, minimum translation was restricted to a pixel and thus, round-off integer values were obtained for each bubble shift. To trace the shift within a pixel accuracy, interpolation method was implemented using 'polyfit' and 'polyval' functions from 'numpy' library (Figure SI3(e)). A parabolic curve was fitted among five referral points (two on each side of maxima in row and then, in column). The shift in decimals within a pixel was derived from its peak value. This translation was then converted to velocity knowing the frame time from 'fps' and scale (ruler used) used in high speed camera. Similar process was enumerated for each pair of adjacent frames in a complete selected list by programming a loop in python. Finally, all the calculated results of bubbles

translation and velocity was imported in an excel sheet for further post-processing into graphs.

Each pair of adjacent frames (frame time = 0.5 ms) was interpreted for correlation to obtain bubble velocity. While horizontal velocity of printer was interpreted using a pair of frames separated by 20 frames (frame time = 0.01 s). Similar scripts for vertical and horizontal cross-correlation were utilized to trace the bubble translation at different printer velocity and ink flow rates.

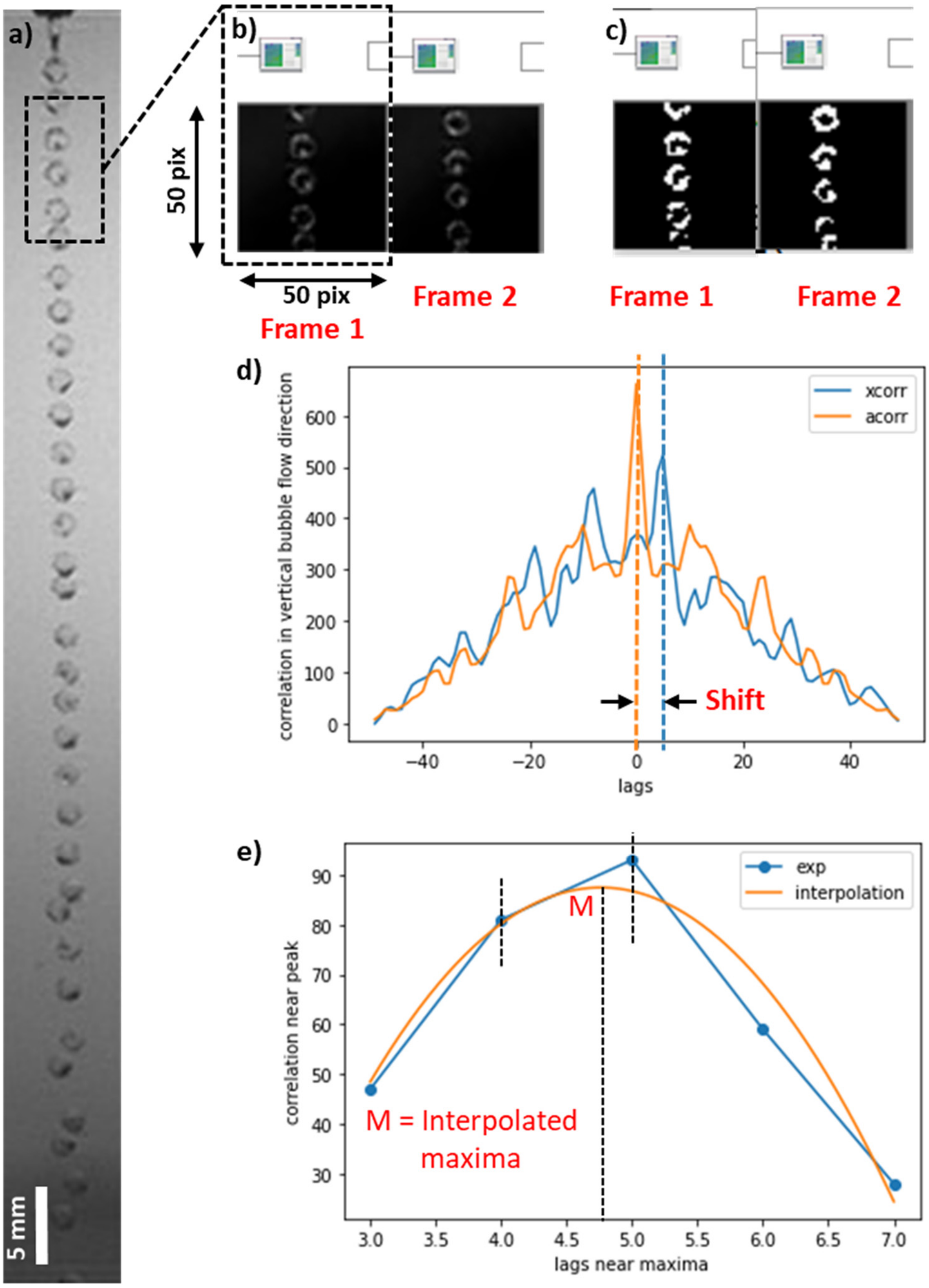

Figure S3: Cross-correlation tool for image processing to calculate bubble velocity. a) High speed video frame of flowing bubbles illustrating the window selected near nozzle to calculate correlation with next adjacent image (not shown). b) Background subtracted and contrast enhanced frame 1 and 2 of 50*50 pixels to highlight the area of interest and reduce the

interference. c) Binary conversion of frame 1 and 2 by using OTSU threshold for cross-correlation scan. d) Correlation function obtained after pixel-by-pixel scan of 2 frames and each row was added (output as column matrix) to highlight the maxima peak. Difference between the 2 maxima peaks, case 1- acorr and case 2- xcorr, (case 2 – case 1 = 5 pixels) indicated the shift of bubbles in 0.5 ms as used to calculate velocity of bubbles. e) The shift of bubbles in d) was restricted to one pixel, therefore, interpolation was performed to determine peak with sub-pixel accuracy. Maximum 'M' of the interpolation function using 5 referral points as shown results in a shift of 4.762 pixels.

In Figure SI4, for executing image processing to detect the average diameter of the printed foam samples, images were cropped and threshold using Otsu's method to eliminate background interference. A morphological transformation from opencv 'cv2.MORPH_CLOSE' was applied onto the images to remove the remaining small pixel interference. This aided in optimally fitting the circle from the detected pixels present at the circumferential positions. Thus, every point on foam was represented by black point and background by white. Each black point coordinate [x,y] was applied to the general equation of circle '$x^2 + y^2$ + ax +by + c = 0' (a,b,c = constant). Solving of this equation returned the center of circle and its mean radius. A circle fit was plotted (using plt library) on each image (for each printer velocity) from the calculated radius and center coordinates. Correspondingly, a theoretical calculation for deviation was designed to compare the results with experiment and summarized in a graph (plt.plot function).

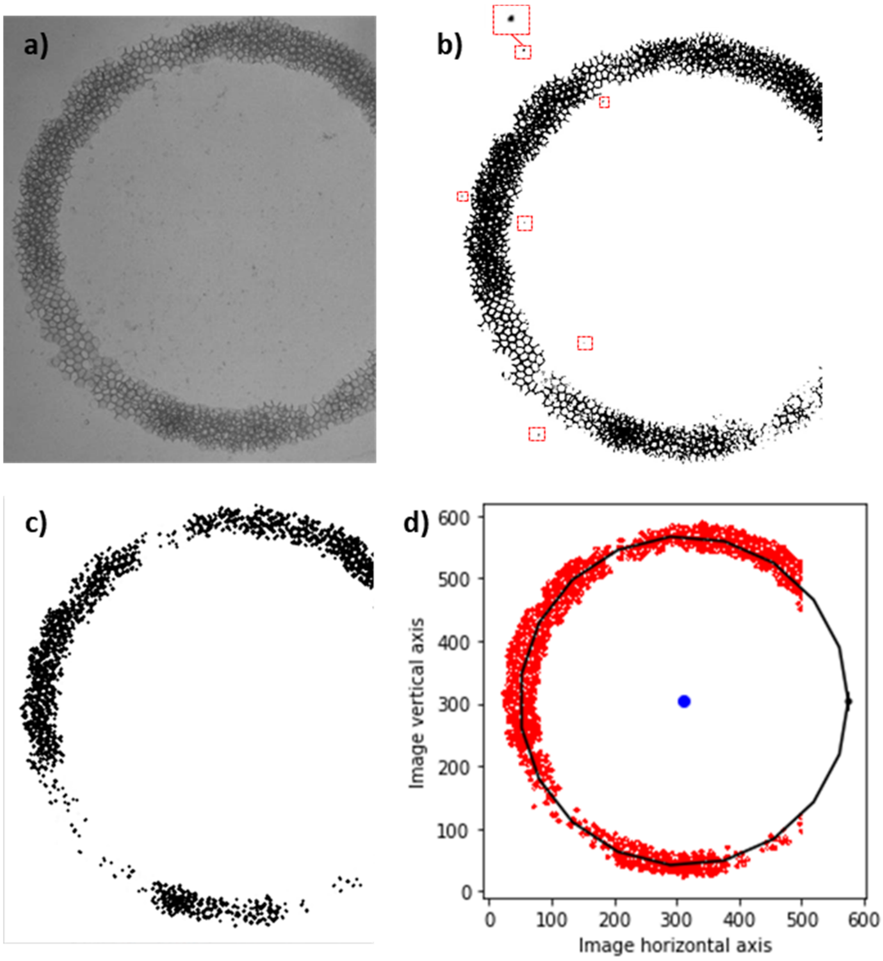

Figure S4. Image processing and circle fit on the printed foam circular samples. a) Foam printed at 100mm/s printhead velocity. b) Conversion of (a) image into binary using OTSU thresholding after background subtraction and contrast enhancement to eliminate noise points. The red enclosed regions represent the noise that was not removed after pre-processing of images. c) Morphological transformation of binary images to remove remaining these noise points. d) Resulting pattern as used to fit a circle (in black) with the center coordinates as shown in blue.

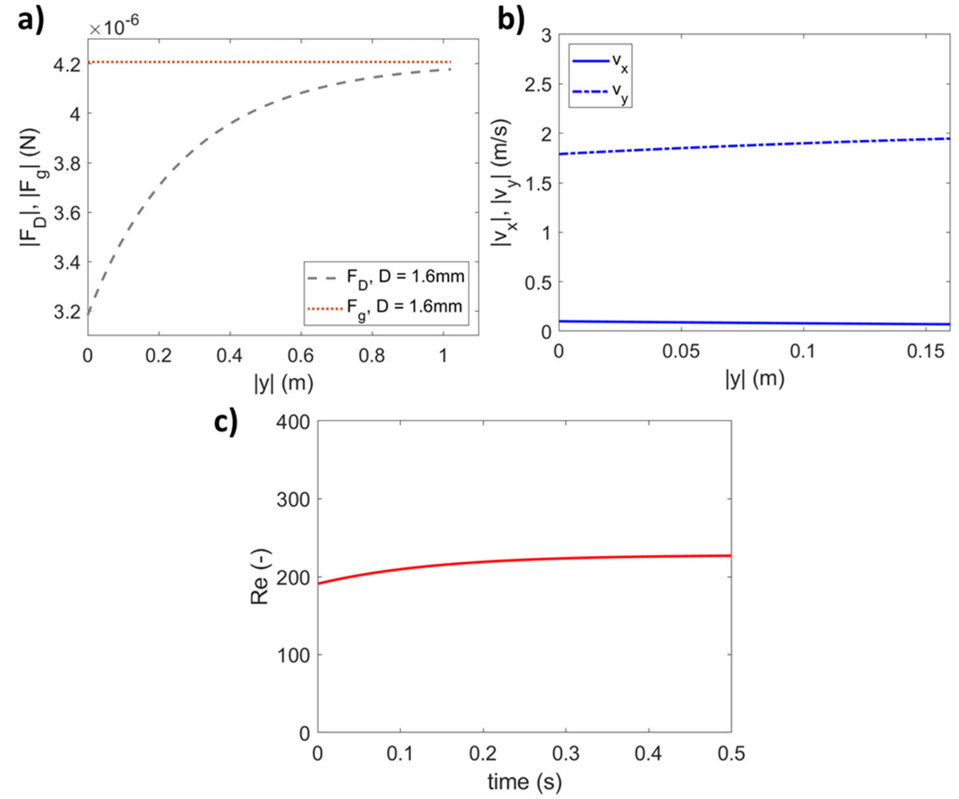

Figure S5: Theoretical analysis of the drag and gravity forces and their influence on the flight of a single bubble. a) The force as a function of the distance from the nozzle for a bubble with a diameter of 1.6 mm. The gray dashed line represents only drag force while the orange dotted line represents the gravity force only. b) Horizontal (v_x) (blue dash-dote line) and vertical (v_y) (blue solid line) velocity of a bubble as a function of the distance traveled from the nozzle including both the gravity and drag forces. c) Behavior of the Reynolds number with time (red line) as the bubble velocity changes during its vertical translation as shown in b).

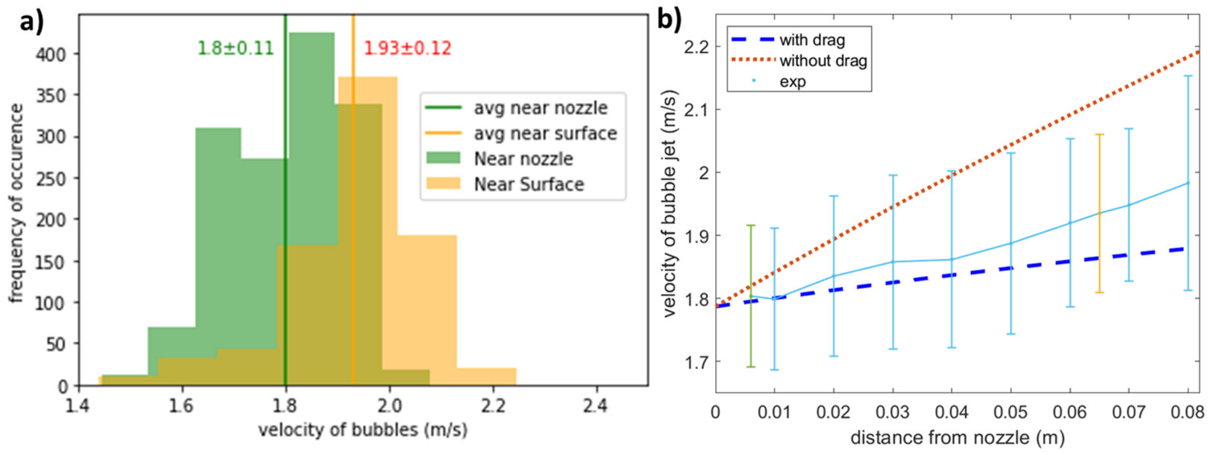

Figure S6: Velocity profile of bubbles in motion as a function of the distance travelled from the nozzle obtained from post-processing of high speed frames. a) Bubble velocity measured after bubble breakup, 6mm from the nozzle (green) and at 65mm from the nozzle (yellow) as indicated by colored bars in Figure 2(a), for Q= 12 ml/min, P=3.5 kPa and printhead velocity 100 mm/s. b) Measured (light blues solid line with error bars) and calculated bubble velocity

without drag (orange-dotted line) and with drag (dark blue dashed line) as a function of the distance from the nozzle. Velocities in both graph are experimentally determined from a cross-correlation of more than 1800 frames. The error bars in both graphs are taken from the standard deviation of the measured bubble velocities.

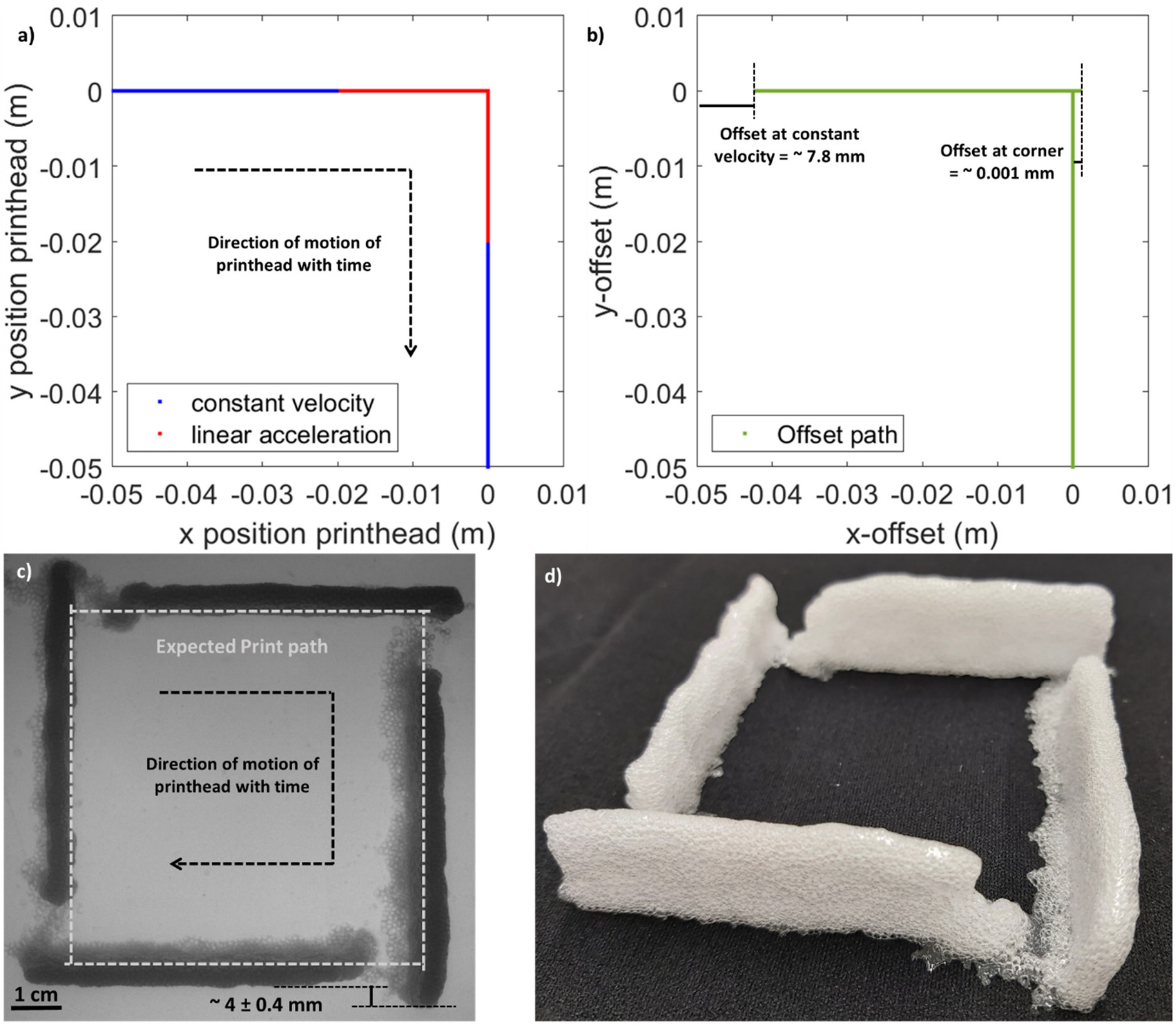

Figure S7: Model and measurements of the printed bubble stream for sharp corners, for a printhead velocity of 200mm/s and acceleration 1m/s^2. (a) The trajectory of the printhead as a function of time, for a sharp turn at corner. Each dot is separated by a time step $\Delta t = 1$ms. (b) The modeled deposition location of the bubble stream for a printhead-build platform distance $h = 10$cm, corresponding to the printhead trajectory in figure (a). (c) Photograph of a printed 3D foam structure in the design path are shown as gray line segments. 25 layers of foam were deposited on top of each other. The white line indicates the modeled print path. The mismatch between the observed path and the modeled result is due to the strong vibrations in the printhead, which are not included in the model. (d) Side view of the 3D printed foam structure shown in (c). (*https://3dprinterly.com/3d-printing-ghosting-ringing-echoing-rippling-how-to-solve/*, accessed on 15 July 2022)

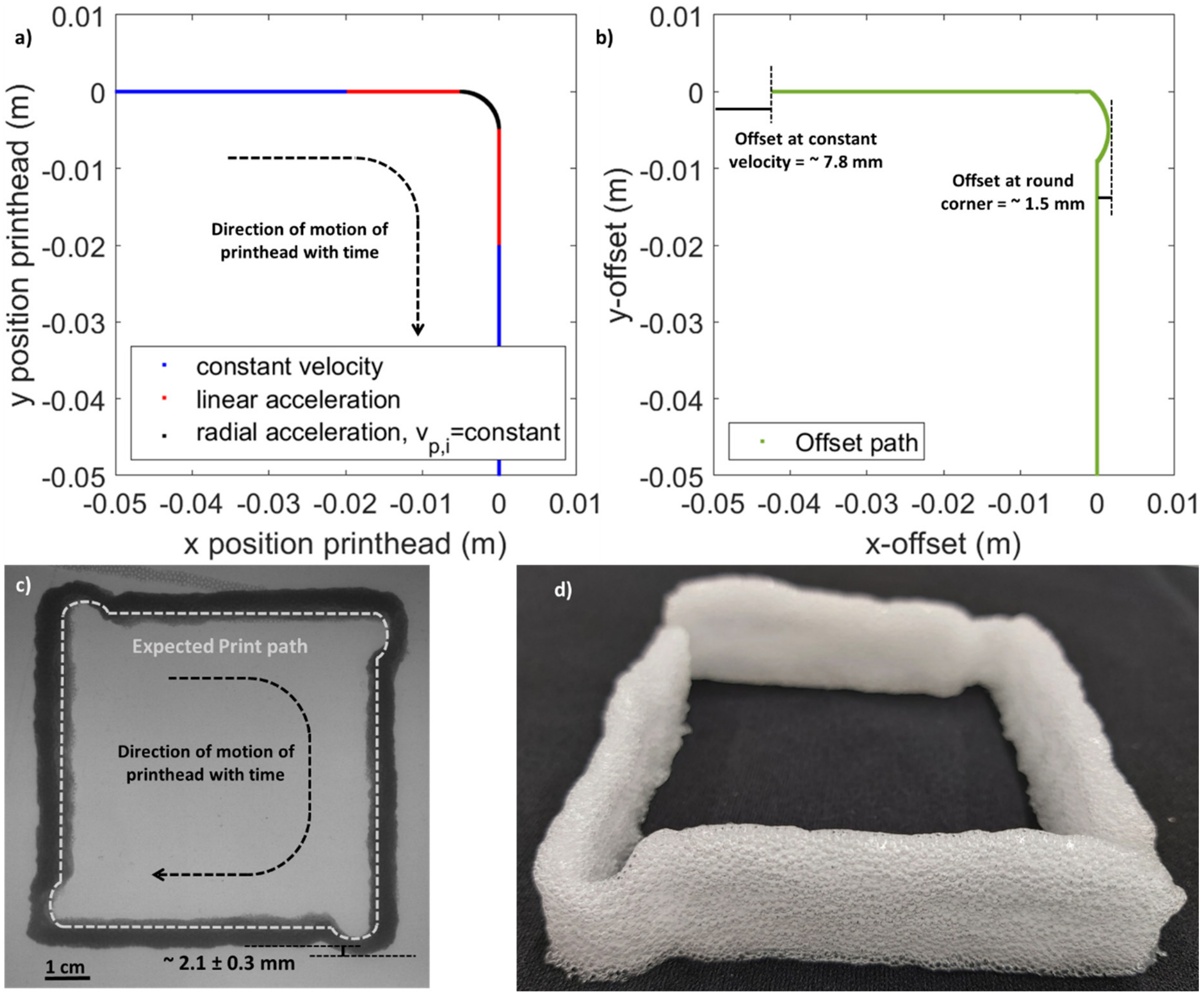

Figure S8: Model and measurements of the printed bubble stream for round corners, for a printhead velocity of 200mm/s and acceleration 1m/s^2. a) The trajectory of the printhead as a function of time, for a smooth and gradual turn at corner. At round corner (black dots), $v_{p,i}$ (instantaneous printhead velocity at end of decelerated region (red dots)) is constant. Each dot is separated by a time step $\Delta t = $ 1ms. b) The modeled deposition location of the bubble stream for a printhead-build platform distance $h = $ 10cm, corresponding to the printhead trajectory in figure (a). c) Photograph of a printed 3D foam structure (top view). The expected print path is shown as a dashed line. 25 layers of foam were deposited on top of each other. d) Side view of the 3D printed foam structure shown in (c).
